# Prenatal Androgenization Induces Anxiety-Like Behavior in Female Rats, Associated with Reduction of Inhibitory Interneurons and Increased BDNF in Hippocampus and Cortex

**DOI:** 10.1155/2019/3426092

**Published:** 2019-06-10

**Authors:** Bojana Rankov Petrovic, Dragan Hrncic, Dusan Mladenovic, Tatjana Simic, Sonja Suvakov, Djurdja Jovanovic, Nela Puskas, Ivan Zaletel, Milica Velimirovic, Valentina Cirkovic, Djuro Macut, Olivera Stanojlovic, Aleksandra Rasic-Markovic

**Affiliations:** ^1^Institute of Medical Physiology “Richard Burian”, School of Medicine, University of Belgrade, 11000 Belgrade, Serbia; ^2^Institute of Pathophysiology, School of Medicine, University of Belgrade, 11000 Belgrade, Serbia; ^3^Institute of Clinical and Medical Biochemistry, School of Medicine, University of Belgrade, 11000 Belgrade, Serbia; ^4^Institute of Histology and Embryology, School of Medicine, University of Belgrade, 11000 Belgrade, Serbia; ^5^Clinic for Endocrinology, Diabetes and Metabolic Diseases, Clinical Center of Serbia, 11000 Belgrade, Serbia

## Abstract

Anxiety is one of the most frequent psychiatric disorders. Despite the fact that most studies describe an anxiolytic effect of testosterone, hyperandrogenemia in mothers is assumed to be related to an increased risk of mood disorders in their offspring. An increasing body of scientific evidence suggests that an altered expression of interneuronal markers of the hippocampus may be the cause of anxiety. The aim of this study was to examine the influence of maternal hyperandrogenemia on behavioral parameters of anxiety-like behavior, neuropeptide Y (NPY) and parvalbumin (PV) expression in the hippocampus, and the level of the brain-derived neurotrophic factor (BDNF) in the hippocampus and cerebral cortex. Pregnant female Wistar albino rats were treated with testosterone undecanoate on the 20th day of gestation. Anxiety-like behavior in adult female offspring was evaluated by the elevated plus maze test and the open field. The number of PV and NPY immunoreactive cells in the hippocampus was determined immunohistochemically. The level of BDNF expression in the hippocampus and cerebral cortex was analyzed with the Western blot test. Prenatal hyperandrogenization increased anxiety-like behavior in female offspring and decreased expression of NPY+ and PV+ in the CA1 region of the hippocampus as compared to the control group. BDNF expression in the hippocampus and cerebral cortex of prenatally androgenized female offspring was significantly increased in comparison with the controls. Prenatal hyperandrogenization may be the cause of anxiety-like behavior in female offspring. Decrease in NPY and PV expression in the hippocampus may explain the possible mechanism of hyperandrogenization induced anxiety.

## 1. Introduction

Genetic sex predisposes the endogenous hormonal milieu which further on causes fine variances in the structure and functioning of the central nervous system [1]. Most clinical studies have concluded that women have an increased overall sensitivity to anxiety in comparison to males [2]. Imaging techniques have revealed these differences in anxiety-relevant brain regions: hippocampus, the amygdala complex, and prefrontal cortex (PFC) [3]. Male/female differences in hippocampal morphology are small but have been consistently proven in the CA1 region [4]. A larger hippocampus in males is evident in early life and correlates with larger number of neurons and glial cells [5, 6]. Much of these is ascribed to effects of testosterone through induction of spines and spine synapses on the dendrites of CA1 pyramidal neurons, as well as alterations in long-term synaptic plasticity (LTP) and hippocampally dependent cognitive behaviors [7]. How these effects occur remains largely unknown.

A vast majority of clinical and animal studies have described exogenous testosterone also as an anxiolytic agent in males and an anxiogenic agent in females [8–10]. Hormonal fluxes in the mother during critical prenatal periods can induce long-term effects on brain growth which could result in altered behavior and increased susceptibility to chronic disease, such as metabolic and psychiatric disease [11]. Women with polycystic ovary syndrome (PCOS) exhibit high circulating androgen levels during pregnancy [12], which is assumed to be related to an increased risk of mood disorders in their offspring [13].

There has been a growing interest in the role of inhibitory interneuron networks in anxiety and mood disorders [14]. They provide well-timed inhibitory input that dictates the temporal window for synaptic excitation and subsequent action potential initiation, thus controlling the timing of information flow [15]. It has been shown that the activity of PV+ and NPY+ interneurons plays a role in maintaining normal cognitive function, circadian rhythms, food intake, and anxiety [16–18]. Specific subtypes of NPY+ cell types are found to be stress-sensitive, which lead to impairment of endogenous NPY release and alteration in CA1 circuit function which altogether potentially contribute to heightened anxiety [19]. GABA-ergic interneurons expressing the calcium binding protein PV makes approximately 24% of interneurons in the CA1 region [20]. The activity of PV+ interneurons is known to drive many essential behaviors [21, 22], and proper control of PV interneurons activity in the dentate gyrus (DG) is critical for regulation of the anxiety, social interaction, and fear extinction [23].

The brain-derived neurotrophic factor (BDNF) has been implicated in the regulation of cell growth, cell differentiation, and synaptic modification and is highly expressed in the developing and adult hippocampus [24, 25]. Rodent studies have shown that testosterone withdrawal in males subsequent to orchiectomy decreases BDNF within the hippocampus, as well as in certain motoneurons [26, 27]. Additionally, certain studies have found that hyperandrogenemia in women with PCOS could correlate to increased levels of the BDNF [28]. Furthermore, it has been demonstrated that alterations in BDNF expression may affect anxiety-related behavior [29], although the neural circuitry involved in these processes remains to be exactly defined. A single nucleotide polymorphism (SNP) of the coding region of the BDNF gene (Val66Met) has been identified as a risk factor for anxiety disorders, including posttraumatic stress disorder [30].

The relation between BDNF, the survival of inhibitory interneurons and anxiety is still not established. Based on this background the aim of the present study was to determine the effects of prenatal androgenization on anxiety-like behavior, NPY and PV immunoreactivity in the hippocampus, and BDNF levels in the hippocampus and the cerebral cortex in female offspring. Possible association could imply potentially therapeutical usage of BDNF in anxiety.

## 2. Materials and Methods

### 2.1. Experimental Procedure

Female Wistar rats (n = 10, age: 75-90 days, body weight: 180-200 g) were obtained from the vivarium of the Military Medical Academy, Belgrade, Serbia. Individual pairs of male and female rats were kept overnight in a polycarbonate cage (55x35x30 cm) in standard animal housing conditions (temperature: 21–22°C; humidity: 55-65% and 12-hour light/dark cycle). Day 1 of gestation was taken to be the day when a sperm-positive vaginal smear was observed [31]. Pregnant dams were then individually housed in different cages and were kept in standard environmental conditions. They were randomly divided into two groups: experimental, testosterone treated (n=5) and control (n=5). On the 20^th^ day of gestation experimental rats were treated with testosterone undecanoate 100 mg/kg (Nebido®, Bayer, 1000 mg/4 ml) and the control group was treated with castor oil. The substances were given subcutaneously (s.c.). The time and dosage of testosterone injection were set according to the studies of Teherani et al. and Callies et al. [32, 33]. Embryonic day 20 was chosen for testosterone undecanoate administration, since this is known as a model of PCOS, without morphological disturbances [32]. We used testosterone undecanoate in a dose of 100 mg/kg, since it has been shown that this concentration maintains physiological level of testosterone in male rats for a minimum of four weeks [33] and so it is suitable for induction of prenatal androgenization in female rats [34]. Pups were housed with their mothers in the same cage and under the same laboratory conditions until postnatal day 21 (PN). Female offspring of experimental (T; n=12) and control groups (C; n=12) were kept in standard laboratory conditions with* ad libitum* food and water. To avoid the effect of long-term social isolation, animals were housed in groups (2-3 per cage). Animal handling and behavioral testing were carried out during the bright phase of the day-night cycle, between 70 and 85 days of age. All experimental procedures were in accordance with the European Parliament Directive (86/609/EEC) and were approved by the Animal Care Committee of the University of Belgrade (license number 323-07-06141/2015-05/11).

### 2.2. Behavioral Testing

Anxiety-like behavior and locomotor activity testing was carried out at 70-85 days of age, during the bright phase of the day-night cycle. Female offspring of C and T groups (12 per group) were tested in the diestrus phase of the estrous cycle, which was confirmed by vaginal smears.

#### 2.2.1. The Elevated Plus Maze (EPM)

The EPM test is used to assess anxiety-related behavior in rodent models of CNS disorders [35]. The EPM comprised two open and two closed arms (50x10 cm) enclosed by 30 cm high walls. Rats were placed in the junction area and were allowed to explore the maze for 5 min. The behavior and movement of each animal was recorded by a camera (*HicVision Bullet 2612*) and subsequently scored by a blinded experimenter. The analyzed behavioral parameters were (a) time spent in the open arms, (b) the number of open arm entries, and (c) the number of head dipping. Anxiety was indicated by the time spent in the open arms and the number of open arm entries. An arm entry was defined as the entry of all 4 limbs into that arm. After each trial, the arms were cleaned with 70% ethanol and dried using paper towels.

#### 2.2.2. Open Field Test (OF)

The OF test is used for assessing both exploratory and locomotor activity in the open field apparatus. The pattern of exploration can also be used as an index of anxiety, since anxious animals tend to spend more time in the periphery. Locomotor activity was tested immediately after EPM for 30 min in photo-cell equipped activity boxes. An automated system fully equipped with infrared sensors (*Experimetria Ltd., Budapest, Hungary*) and its accompanying software (*Conducta 1.0*) was used to monitor rat behavior. The system registers the horizontal and vertical activity of animals. A sound-attenuated area (48x48 cm) with 12 lux red lighting was surrounded by black walls, 40 cm high. The whole area was divided into 16 squares with the 4 middle squares marked as the central area. The time spent in the center and the number of entries into the center was recorded as the measure of anxiety-like behavior. The total ambulatory distance was recorded as an index of locomotor activity. The open field arena was cleaned with 70% ethanol between trials.

After behavioral testing, the animals were anesthetized by intraperitoneal application of ketamine (10 mg/kg) and xylazine (5 mg/kg) and then sacrificed by decapitation. Trunk blood samples were collected for serum sex hormones assays, while brains were rapidly removed from the skull for further histological and Western blot analysis. Blood sampling and tissue collection was done during the diestrus phase of the estrous cycle.

### 2.3. Immunohistochemistry

After sacrificing the rats, brains were carefully removed from the skull, fixated in 4% paraformaldehyde in 0.1M phosphate buffer, pH=7,4 and embedded in paraffin. For immunohistochemistry, 5 *μ*m thick coronal brain sections were dewaxed, rehydrated, and treated with citrate buffer (pH 6.0) in a microwave for antigen retrieval. Endogenous peroxidase activity was blocked with 3% H_2_O_2_, followed by 1-hour incubation in normal goat or horse serum. In the next step, slices were incubated in rabbit polyclonal anti-NPY antibody (1:250,* AbD Serotec*) and mouse monoclonal anti-PV antibody (1:1000,* Sigma-Aldrich*) overnight at room temperature. Sections were further incubated for 1 hour in biotinylated anti-rabbit secondary or biotinylated anti-mouse secondary antibody, followed by ABC-complex (VECTASTAIN Elite ABC HRP Kit, Vector Laboratories). Visualization of the immunoreactive sites was done by 3,3′-diaminobenzidine chromogen (DAB Peroxidase (HRP) Substrate Kit, Vector Laboratories). Finally, sections were counterstained with Mayer's hematoxylin and covered. The staining specificity was checked by omitting the primary antiserum. No immunoreactivity was detected in these sections. Image capturing of NPY, PV stained hippocampal slices was done on Leica DM4000 B LED microscope with digital camera Leica DFC295 and by using Leica Application Suite (LAS, v4.4.0) software system. The surface area of each of the hippocampal regions (CA1, CA2/3, DG) in the chosen sections was measured by the same software system and the number of NPY and PV immunoreactive cells was counted in each of those areas, after which the number of immunoreactive neurons was expressed per 1 mm^2^ of investigated region in order to standardize the number of counted cells [36]. The counting was always done on the dorsal hippocampus on animals from C and T groups (6 per group). The counts were made by independent experimenters who were blind to the experimental protocol.

### 2.4. Western Blotting for BDNF

Isolated hippocampi and cortices from C and T groups (6 per group) underwent homogenization in 8 volumes of the standard RIPA buffer containing the protease inhibitor cocktail (*Sigma, Germany*). Tubes were centrifuged at 14,000 rpm for 30 minutes at 4°C, and supernatant was collected. All samples were stored at -80°C until further analysis. Protein concentrations were determined by the Bicinchoninic Acid (BCA) Protein Assay (*Sigma, Germany*). A total of 50*μ*g of proteins was loaded on 15% polyacrylamide gel and electrophoresis was performed for 90 minutes at 150V. After wet transfer, nitrocellulose membranes were blocked in 5% low-fat milk (*Carl Roth, Germany*) for 1 hour at room temperature and then incubated with primary antibodies: rabbit polyclonal anti-BDNF (1:2000;* Millipore, USA*) and mouse monoclonal anti-B actin (1:3000,* Sigma, Germany*) overnight at 4°C. Membranes were washed in TBS-Tween and incubated with appropriate secondary antibodies: anti-rabbit (1:2000,* Amersham*,* UK*) and anti-mouse (1:3000,* Dako, USA*) for an hour at room temperature. BDNF and pro-BDNF protein bands were detected at 18kDa and 30kDa, respectively, using the Clarity Western ECL Substrate (*BioRad, USA*) by ChemiDoc (*BioRad, USA*).

### 2.5. ELISA for Hormones

Blood samples from C and T groups (8 per group) were obtained between 11a.m. and 1p.m. using Vacutainer plastic blood collection tubes after an overnight fast with the purpose of reducing circadian alterations in adhesion molecules that may be potentially associated with cortisol release or food intake. Their blood was collected in serum separator tubes. The tubes were inverted 5 times and allowed to clot for 30min at room temperature, followed by another 30min at 4°C. Serum and blood cells were separated by centrifugation (15min, 3000rpm). Serum samples were stored at −80°C. Testosterone concentrations were measured using the Rat Testosterone (T) enzyme-linked immunosorbent assay (ELISA) Kit (*Cusabio, Houston, USA*), in serum samples in accordance with the manufacturer's instructions. Assay sensitivity was < 0.06ng/ml. The standard curve range was from 0.13 to 25.6ng/ml. The results were calculated with the standard curve using “Curve Expert 1.4”. Estradiol concentrations were measured using the Rat Estradiol (E2) ELISA Kit (*Cusabio, Houston, USA*), in serum samples following the manufacturer's instructions. The detection range was 40pg/ml-1400 pg/ml. The results were calculated in comparison with the standard curve using “Curve Expert 1.4”. Each sample was run in duplicate.

### 2.6. Estrous Cycle

In laboratory rats the estrous cycle lasts approximately 4-5 days and consists of four stages known as proestrus, estrus, metestrus, and diestrus. Proestrus lasts approximately 12 h, and during this phase, estradiol levels increase rapidly, stimulating gonadotropin release and ovulation. Estrus duration ranges between 24 and 48 h, and ovulation usually occurs during this phase. Proestrus and estrus comprise the follicular phase of the estrus cycle. It is well documented that metestrus is a short stage of 6–8 hr in rats. In the absence of mating at the time of ovulation, the corpora lutea are formed and they secrete progesterone. Progesterone is secreted by the corpus luteum during metestrus and declines during diestrus. Diestrus is the longest stage of the estrus cycle with an average duration of 48–72 h. During diestrus serum hormone levels are much lower [37].

Phases of the estrous cycle can be detected by examining the vaginal smear. The predominant feature of the proestrus is the presence of nucleated epithelial cells, often seen in clusters or strands. Estrus is routinely identifiable by the presence of large numbers of needle-like, anucleated, cornified epithelial cells. Metestrus smear is characterized by a combination of leukocytes and cornified and rounded epithelial cells, while diestrus smear can often be almost exclusively leukocytic [38].

### 2.7. Statistical Analysis

All analyzed parameters were tested for normality of the data using the Kolmogorov-Smirnov test. For normally distributed data, the significance of the differences was evaluated by one-way ANOVA while for nonnormally distributed data the nonparametric Mann-Whitney* U* test was used to determine the significance of the differences between the groups. The significance was considered statistically significant if p < 0.05. Statistical analysis was performed with the SPSS version 17.0 statistical package (*IBM SPSS Statistics*).

## 3. Results

### 3.1. Behavioral Testing

#### 3.1.1. Elevated Plus Maze

**Figure 1:**
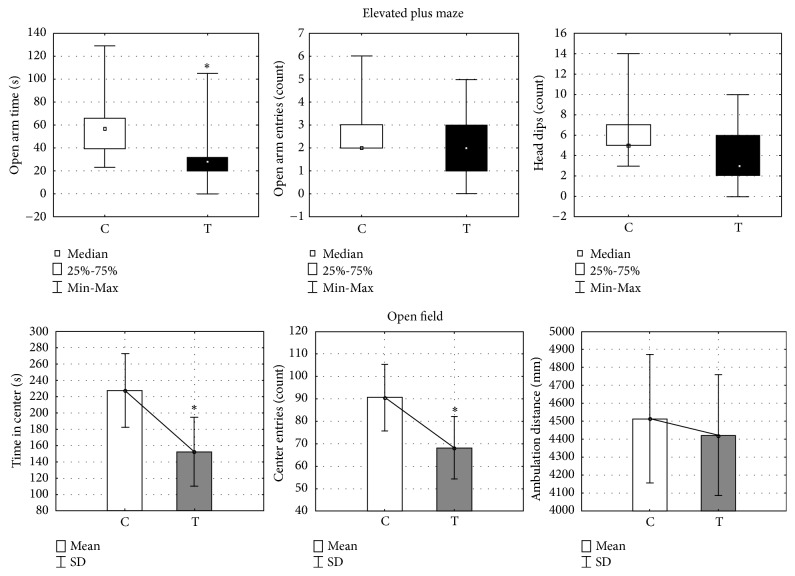
Open field and elevated plus maze activity in female offspring prenatally treated with testosterone undecanoate. Testosterone undecanoate (100 mg/kg, s.c.) was administered to female rats on gestational day 20 and their offspring was tested between postnatal days 70 and 85. Scores on elevated plus maze activity: (a) time spent in the open arms, (b) number of entries into the open arms, and (c) number of head dipping. Boxplots represent medians ± quartile range, n=12. Scores on open field activity: (a) time spent in the center, (b) number of entries into the center, and (c) total ambulatory distance. Bars represent means ± SD values, n=12. The significance of the difference was estimated by Mann-Whitney* U* test or one-way ANOVA, respectively (*∗*p < 0.05). C, control group; T, testosterone group.

Prenatal treatment with testosterone undecanoate significantly reduced time spent in the open arms as compared to the control group (U=25.5, Z=2.17, p < 0.05) (Figure 1), while the number of entries in the open arms was not affected by T treatment (U=37.5, Z=1.37, p> 0.05). Exploratory activity in the EPM, expressed by the amount of head dipping, was not significantly different when compared with the control group (U=32.0, Z=1.74, p > 0.05) (Figure 1).

#### 3.1.2. Open Field

Specific manners of locomotor activity in the open field test were differently expressed in the T and C group. The indicators of anxiety-like behavior, such as time spent in the center (F=6.77, p < 0.05) and the number of entries into the center (F=5.4, p < 0.05), were significantly reduced in the T group as compared to the C group (Figure 1). The total ambulatory distance used as the index of locomotor activity was not significantly different between the groups (F=16660, p > 0.05, Figure 1).

### 3.2. Immunohistochemistry

**Figure 2:**
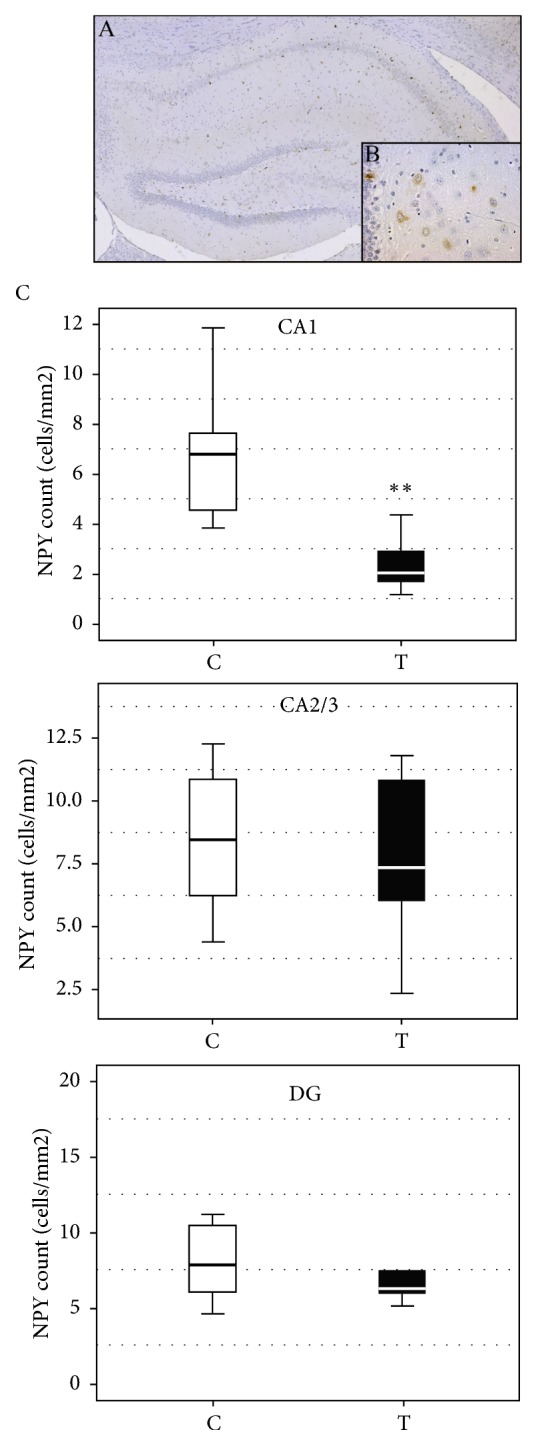
(A, B) Representative caption of immunohistochemical expression of NPY+ interneurons in the rat hippocampus from the control group. (A) Hippocampus of control group, magnification 50x; (B) NPY immunoreactive cells in control group, high magnification 400x. (C) The effect of prenatal testosterone undecanoate treatment on the number of NPY immunoreactive interneurons in the hippocampal regions. Boxplots represent medians ± quartile range, n=6. The significance of the difference was estimated using Mann-Whitney* U* test (*∗∗*p < 0.01). For details see caption of Figure 1.

Immunohistochemical staining with antibodies against NPY and PV allowed examination of the distribution of GABA-ergic interneurons in the hippocampal sections CA1, CA2/3, and DG in the offspring of both the testosterone treated and the control female offspring group. NPY immunoreactive neurons in the hippocampus were predominantly located in the pyramidal cell layer of the CA1 and CA2/3 regions, whereas, in the DG, the NPY immunoreactive neurons were expressed mostly in the hilus (Figures 2(A) and 2(B)). Prenatal testosterone treatment resulted in a significant decrease in the number of NPY immunoreactive neurons in CA1 as compared to the control group (U=16.0, Z=3.86, p< 0.01; Figure 2(C)), while there were no differences in the number of NPY in the CA2/3 (U=103.0, Z=0.85, p > 0.05; Figure 2(C)) and the DG (U=112.0, Z=0.81, p > 0.05; Figure 2(C)).

**Figure 3:**
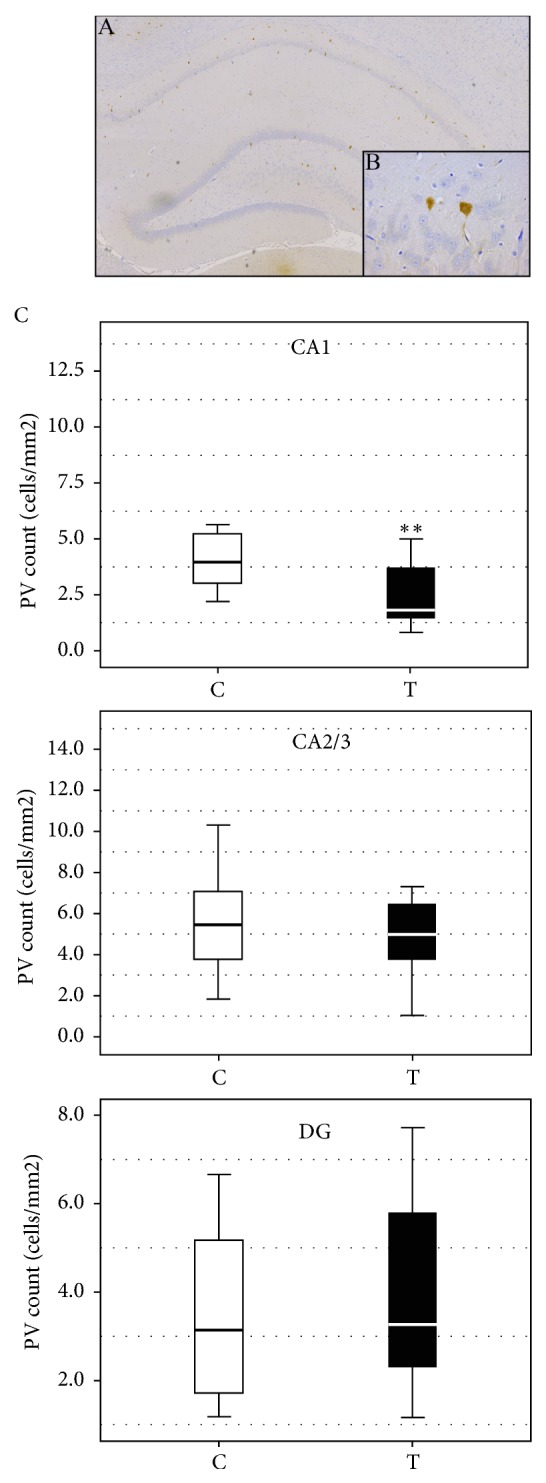
(A, B) Representative caption of immunohistochemical expression of PV+ interneurons in rat hippocampus from the control group. (A) Hippocampus of control group, magnification 50x; (B) PV immunoreactive cells in control group, high magnification 400x. (C) The effect of prenatal testosterone undecanoate treatment on the number of PV immunoreactive neurons in the hippocampal regions. Boxplots represent medians ± quartile range, n=6. The significance of the difference was estimated using Mann-Whitney* U* test (*∗∗*p < 0.01). For details see caption of Figure 1.

Parvalbumin interneurons of both groups were located mostly within, or in the vicinity of the pyramidal cell layer in the CA1 and CA2/3, and mostly in the granular cell layer in the DG (Figures 3(A) and 3(B)). PV immunoreactivity was expressed in the same manner as NPY. The expression of PV in the CA1 region was statistically significantly lower in the testosterone group than in the control group (U=46.0, Z=3.02, p < 0.01, Figure 3(C)). There was no statistically significant difference in PV expression in the CA2+3 (U=108.0, Z=0.66, p > 0.05; Figure 3(C)) and the DG regions between the groups (U=117.0, Z=0.91, p > 0.05, Figure 3(C)).

### 3.3. Western Blot Analysis for Protein Expression of BDNF

**Figure 4:**
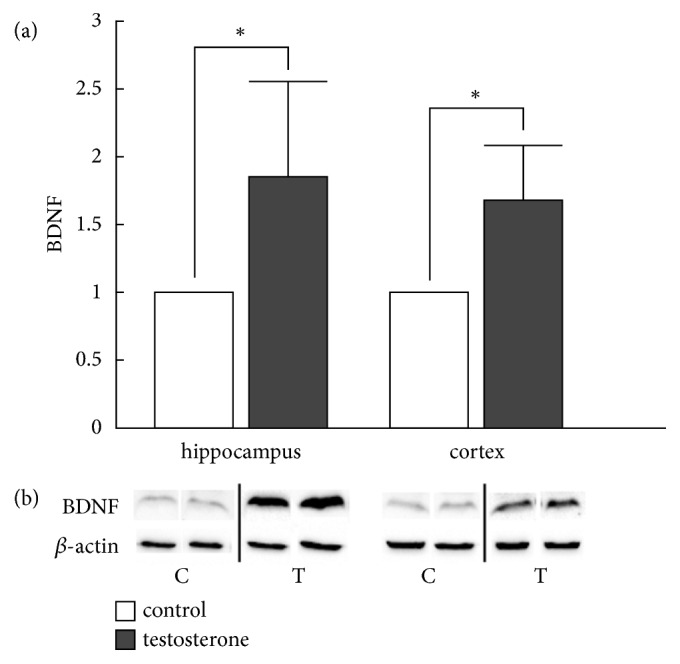
The effect of testosterone administration on BDNF protein (18kDa) expression in the rat hippocampus and cortex. (a) BDNF protein expression in the hippocampus and the cortex of female offspring was significantly increased in the testosterone group, as compared to the control group. BDNF expression in the control group was standardized to 1 (100%) (b) Representative blots present nonadjacent bands originating from the same gel. Densitometry data are presented as mean ± SD values, n=6, *∗* p < 0.05. For details see caption of Figure 1.

**Figure 5:**
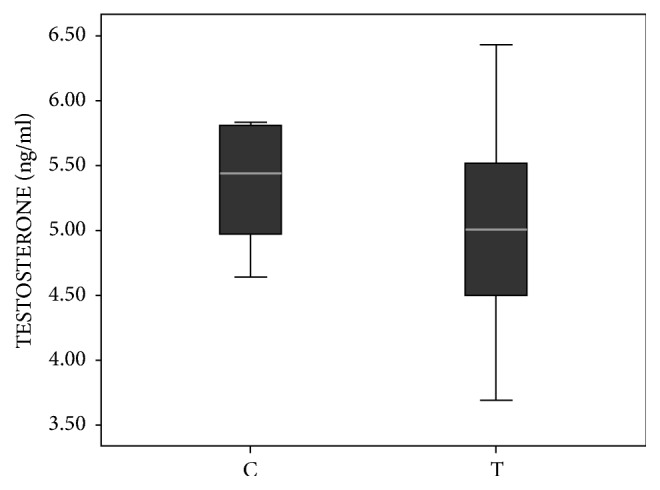
Concentrations of testosterone in serum of prenatally androgenized female offspring. The difference between concentrations of testosterone between the groups was calculated using the Mann-Whitney* U* test. Boxplots represent medians ± quartile range, n=8. For details see caption of Figure 1.

Western Blot analysis demonstrated a region-specific increase in mature BDNF expression. BDNF protein expression levels in the hippocampus of female offspring prenatally treated with testosterone revealed a significant increase when compared to the controls (p < 0.05, Figure 5). The expression of the BDNF protein in the cortex showed this same alteration in the examined groups, with a significantly higher expression in the testosterone group when compared to the control (p < 0.05, Figure 4).

### 3.4. Hormonal Levels in the Serum

**Figure 6:**
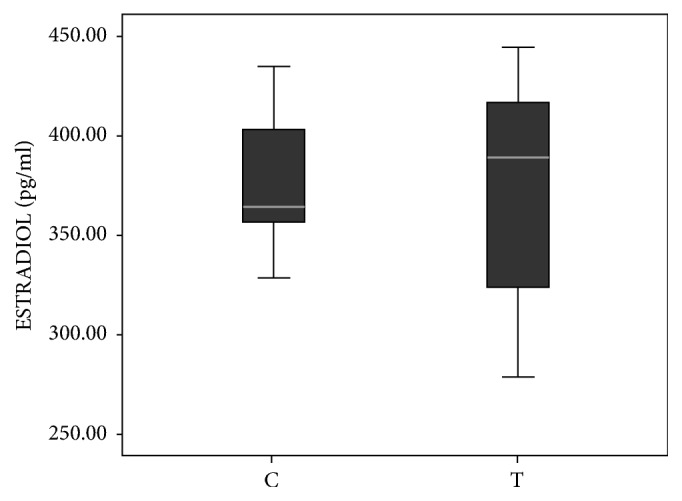
Serum level of estradiol in prenatally androgenized adult female offspring. The difference between concentrations of estradiol between groups was calculated using the Mann-Whitney* U* test. Boxplots represent medians ± quartile range, n=8. For details see caption of Figure 1.

Concentrations of testosterone in the serum of prenatally treated female offspring were not significantly different when compared to the controls (p > 0.05, n=8; Figure 5). The same holds true for estradiol, where a difference in concentration was not found between the groups (T versus C, p > 0.05, Figure 6).

## 4. Discussion

Our results clearly showed that hyperandrogenemia in pregnant dams induced anxiety-like behavior in female offspring later in their life. Immunohistochemical analysis revealed that prenatal androgenization reduced the number of PV+ and NPY+ neurons in the CA1 region of the hippocampus. Additionally, this group of animals also had increased BDNF protein expression levels in the hippocampus and the cortex. Finally, exposure to exogenous testosterone on embryonic day 20 had no effect on circulating levels of testosterone and estradiol in adult female offspring.

Anxiety disorders are amongst the most common of all psychiatric disorders [39] and the role of gonadal steroids in their development is quite complex. Higher prevalence of anxiety in females and hypogonadal men compared with otherwise healthy men points at the anxiolytic effect of androgens [3]. This has been also confirmed in experimental studies, since administration of testosterone decreases anxiety-like behavior in orchiectomized and intact male rats and mice [40, 41]. In contrast to orchiectomized male rats, administration of physiological levels of testosterone had no effect on anxiety-like behavior in ovariectomized female rats [42]. On the other side hyperandrogenemia is the most prominent metabolic feature of PCOS, a common disorder found in women of reproductive age, which can affect psychological profile of the patient as well as of her offspring. Almost 60% of women with PCOS are diagnosed with at least one psychiatric disorder such as anxiety, depression, or an eating disorder [43]. The results of the present study are in accordance with other studies [10, 44] which have also shown that maternal hyperandrogenemia results in increased anxiety-like behavior in female offspring. The mechanisms of anxiogenic effect of prenatal hyperandrogenemia are not completely established. It is known that steroid hormones exert substantial epigenetic influence on early brain development, thus laying the foundation for later interaction between the genome and the environment in creating variations in neural and behavioral phenotypes [45, 46]. Hyperandrogenemia of the mother has been proven to influence the offspring in aspects of genomic action of testosterone during critical periods of development [47, 48]. This indicates that prenatal exposure to high concentrations of testosterone through epigenetic silencing or enhancing of gene expression may influence the development of neural networks and impose the risk of anxiety-like behavior later in life. Another possible mechanism of anxiogenic effect of prenatal hyperandrogenemia, not mutually exclusive from the first one, could be the reduction of the number of PV+ and NPY+ neurons in the CA1 region of the hippocampus. This hypothesis is based on our results which confirmed the association of prenatal androgen treatment with anxiety and reduced NPY and PV immunoreactivity in CA1 hippocampal region. Our findings are in accordance with the study by Joksimovic et al. [36] which has shown a decreased number of NPY interneurons after treatment with the synthetic androgen, nandrolone decanoate, to correlate with anxiogenic and depressive behavior. Furthermore, CA1 hippocampal region is highly susceptible to androgen influence during development [48] and expresses high level of androgenic receptor mRNA [36].

Rodent studies have provided numerous evidences proving the relation between NPY and PV neurons, and anxiety. NPY deficiency is associated with an anxiogenic phenotype and highly anxious rats are more sensitive to the anxiolytic actions of NPY [49, 50]. Additionally, intracerebroventricular administration of NPY decreases anxiety-like behavior in the EPM, Vogel's drinking conflict test and other operant conflict tasks [51, 52]. Site-specific studies have identified the amygdala, locus coeruleus, lateral septum, and hippocampus as regions involved in the anxiolytic properties of NPY [53, 54]. On the other side, optogenetic studies have proven the importance of the hippocampal PV+ interneuronal group in reducing anxiety [15]. PV interneurons are required for synchronization of spiking activity in neuronal networks and subnormal PV interneuron activity is known to affect fear memory [23].

There are limited data in the current literature about influence of prenatal and postnatal hyperandrogenization on BDNF expression in the brain of adult offspring. The nature of this association seems to be very complex and potentially region-dependent. Clinical study conducted in transsexual persons showed that despite dramatic changes in the sex-hormonal milieu BDNF levels was not significantly changed [55]. Some prior studies indicate that gonadectomy and sex steroid replacement do not alter hippocampal BDNF mRNA expression in male adult rats [42] or BDNF protein levels in adolescent mice or aged male rats [56, 57]. These data indicate that testosterone does not seem to play a major role in the regulation of BDNF in females in adulthood. Still, there is evidence to suggest that testosterone is able to modulate brain BDNF levels [58]. Indeed, testosterone increases BDNF and tropomyosin receptor kinase B (TrkB) expression in the adult rodent cortex and songbird higher vocal center [27]. In the forebrain of male mice, increases in BDNF expression corresponded in time to the surge in testosterone at adolescence; and conversely, there was a significant decrease of TrkB, a high-affinity receptor for BDNF, with increased testosterone in the cortex [56]. Based on these and the results of the present study we can assume that androgenization may have different effects on BDNF level in prenatal and postnatal period. This possibility is further supported by different mode of function and different effects of various neurotransmitters in developing versus mature brain.

BDNF is expressed in the cortex and hippocampus and plays an important role in the development, survival, and maintenance of neurons, as well as in the regulation of synaptic plasticity [57]. However, the effects of BDNF may be region-specific. Downregulation of BDNF was found to be associated with Alzheimer's disease, Parkinson's disease, schizophrenia, and possibly depression [58–60]. The present study found an association between increased BDNF in the cortex and hippocampus and the reduction of PV+ and NPY+ interneurons in CA1 region. There are few possible explanations for this finding. First, BDNF level may be increased as an adaptive response of the brain to the reduction of inhibitory neurons caused by prenatal androgenization. Second, BDNF may have no influence on inhibitory interneuron development in the hippocampus and this association is coincident. This hypothesis may be supported by the study of Danzer and McNamara [61], which showed that BDNF was not expressed by hippocampal interneurons. However, this finding is also controversial to some extent. Glorioso et al. [62] found that interneurons express TrkB and require BDNF synthesized by pyramidal neurons for differentiation and maturation. It has also been reported that approximately 80% of PV neurons express TrkB [63]. BDNF has been shown to modulate autoregulatory circuit between excitatory pyramidal cells and inhibitory interneurons in the hippocampus [64]. Based on these findings, it is more probable that BDNF increase in the hippocampus may be considered as an adaptive response.

The effect of BDNF on NPY neurons is even more blurred. The stimulation of Y2 receptors by NPY has been shown to increase BDNF level associated with improvement of motor function in a mouse model of Huntington disease. On the other side, BDNF protein has been found to potentiate the differentiation of inhibitory NPY and PV interneurons in the culture of rat embryos [65]. Furthermore, Purves-Tyson et al. [66] suggest that there are dynamic relationships between BDNF/TrkB and interneuron markers that are dependent on the presence of testosterone. These findings are not consistent with the results of the present study, which showed an inverse relation between BDNF level and the number of NPY neurons. This indicates that NPY neuron development is regulated in a more complex manner with involvement of numerous neurotrophic factors, which may have even different effects in various stages of brain development. Despite all controversies, the results of the present study clearly show that NPY and PV inhibitory neurons in CA1 region of hippocampus are resistant to the effects of BDNF after prenatal androgenization and that BDNF does not support the survival of inhibitory interneurons in this region.

Prenatal testosterone administration had no effect on NPY or PV immunoreactivity in DG. It can be expected, since BDNF was found to induce the differentiation of neural progenitor cells into excitatory granular neurons in DG, not to inhibitory. BDNF and its receptor TrkB may play key roles in regulating the information flow through circuits from the prefrontal cortex, through the CA3 region to the CA1 region, via their mossy fiber axons. This pathway contains the highest levels of the BDNF protein in the CNS [67], which may at least partially explain the findings of the present study of a parallel increase in the levels of the BDNF in the cortex and the hippocampus of prenatally androgenized female rats.

Results of the studies examining the effect of prenatal testosterone on adult sex hormone concentrations are inconsistent. While some studies report that prenatal testosterone treatment increases plasma testosterone levels in adult female offspring [34, 68], other studies report decreased or unchanged plasma testosterone levels in the offspring. In our study neither testosterone, nor estradiol concentration was affected by prenatal testosterone administration. This finding is in accordance with the study of Domonkos et al., which showed that prenatally androgenized females did not exhibit increased plasma concentration of progesterone, estradiol, or testosterone [69]. Furthermore, Hu et al. [10] also showed that prenatal administration of testosterone propionate from the gestational days 15–19 resulted in anxiety-like behavior in adulthood, but also without affecting the levels of serum testosterone in adult female offspring.

Testosterone is lipophilic hormone and it may diffuse across the placenta and exert direct effects on fetal growth and development [70]. However, Vreeburg et al. reported that testosterone infused into the maternal compartment of pregnant guinea-pigs could be detected in fetal plasma only in relatively small amounts [71, 72]. It has been shown that testosterone given to the dam is metabolized or blocked at the placenta. Efficient metabolism of testosterone and androstenedione takes place in placenta which exhibits high activity of 5*α* reductase and 3*β*, 17*β*-hydroxysteroid-dehydrogenases. Furthermore, testosterone in the mother can alter steroid production in the placenta [73], which may affect the fetus. Another potential mechanism by which prenatal testosterone changes behavior of the offspring is the organizational effect which may permanently alter brain morphology and subsequently the behavior.

The novelty of our research was the type of hormone, timing, and duration of exposure. Depo-testosterone preparation was used on the 20th day of gestation, in order to induce constant hyperandrogenemia in pregnant dams. This period is concurrent with the androgen surge in male fetuses of rats (beginning on embryonic day 16 and lasting until embryonic day 21) and this period may be critical for the modulation of female behavior. Previous studies suggest that the exposure of females to androgens during prenatal life may lead to appearance of PCOS phenotype in adulthood [32]. There is a growing awareness that androgenic substances are present in the environment, and it is possible that the female reproductive and metabolic phenotype, as well as behavior, would be altered by androgen exposure, especially during prenatal period.

The strength of the present study is the production of the functional model of late prenatal androgenization without morphological disorders of the reproductive system in female offspring (unpublished data). To the best of our knowledge, this is the first study that examines the association between late prenatal androgenization, anxiety-like behavior, number of NPY+ and PV+ interneurons, and BDNF level in adult female offspring. The anxiety spectrum disorders are not rare in daughters of the mothers with PCOS [74], so decreased number of hippocampal inhibitory interneurons found in this study could give possible explanation for this observation.

One of the limitations of our study is that we have not quantified other important steroid hormones, and we have chosen to test only female offspring. Furthermore, several questions arise from the present study. What is underlying mechanism of decreased NPY and PV expression in adulthood of prenatally treated female rats? Could finding of increased BDNF levels be explained by the epigenetic effect of high prenatal testosterone on BDNF synthesis or the increased levels of BDNF in the hippocampus and cortex are response to decreased number of interneurons in the hippocampus?

## 5. Conclusions

Based on all the aforementioned data, it can be concluded that prenatal androgenization induces anxiety-like behavior in adult female rats associated with decreased number of PV and NPY interneurons in CA1 region of the hippocampus and increased BDNF level in the cortex and hippocampus. Further studies are supposed to bring us answers to aforementioned questions.

## Data Availability

The data used to support the findings of this study are available from the corresponding author upon request.
